# Efficient production of methane from artificial garbage waste by a cylindrical bioelectrochemical reactor containing carbon fiber textiles

**DOI:** 10.1186/2191-0855-3-17

**Published:** 2013-03-13

**Authors:** Daisuke Sasaki, Kengo Sasaki, Atsushi Watanabe, Masahiko Morita, Yasuo Igarashi, Naoya Ohmura

**Affiliations:** 1 Biotechnology Sector, Environmental Science Research Laboratory, Central Research Institute of Electric Power Industry, 1646 Abiko, Abiko-shi, Chiba-ken 270-1194, Japan; 2 Department of Biotechnology, Graduate School of Agricultural and Life Sciences, The University of Tokyo, Yayoi 1-1-1, Bunkyo-ku, Tokyo 113-8657, Japan; 3 Present address: Graduate School of Engineering, Kobe University, 1-1 Rokkodai-cho, Nada-ku, Kobe, Hyogo 657-8501, Japan

**Keywords:** Bioelectrochemical reactor, Supporting material, Carbon fiber textile, Thermophilic methanogenesis, High organic loading rate

## Abstract

A cylindrical bioelectrochemical reactor (BER) containing carbon fiber textiles (CFT; BER + CFT) has characteristics of bioelectrochemical and packed-bed systems. In this study, utility of a cylindrical BER + CFT for degradation of a garbage slurry and recovery of biogas was investigated by applying 10% dog food slurry. The working electrode potential was electrochemically regulated at −0.8 V (vs. Ag/AgCl). Stable methane production of 9.37 L-CH_4_ · L^−1^ · day^−1^ and dichromate chemical oxygen demand (CODcr) removal of 62.5% were observed, even at a high organic loading rate (OLR) of 89.3 g-CODcr · L^−1^ · day^−1^. Given energy as methane (372.6 kJ · L^−1^ · day^−1^) was much higher than input electric energy to the working electrode (0.6 kJ · L^−1^ · day^−1^) at this OLR. Methanogens were highly retained in CFT by direct attachment to the cathodic working electrodes (52.3%; ratio of methanogens to prokaryotes), compared with the suspended fraction (31.2%), probably contributing to the acceleration of organic material degradation and removal of organic acids. These results provide insight into the application of cylindrical BER + CFT in efficient methane production from garbage waste including a high percentage of solid fraction.

## Introduction

Recycling of the huge organic fraction in municipal solid wastes such as garbage and waste from the food industry has been long-awaited (Haruta et al. 2005). Anaerobic digestion using methane fermentation is an effective technology for recovering methane gas as a renewable energy source. It is a low-cost process and produces little residual sludge (Ahring 2003; Forster-Carneiro et al. 2008). Various processes have been exploited to increase the efficiency of methane fermentation. Thermophilic packed-bed systems have been reported to be one of the high-performance reactor designs (Sasaki et al. 2007; Ueno et al. 2007). In the packed-bed system, supporting materials were packed to retain microorganisms and thereby enable operation at a high organic loading rate (OLR) and short hydraulic retention time (HRT; Sasaki et al. 2010a). In our previous study, carbon fiber textiles (CFT) that have surface hydrophobicity and porous structure for better retention of microorganisms were used as supporting materials (Sasaki et al. 2010a).

Previous research has found that a bioelectrochemical reactor (BER) can stimulate microbial metabolism and affect the growth rate of microorganisms by controlling the electron flow in the culture medium (Thrash et al. 2007; Matsumoto et al. 2002). Recently, a BER was used for methane fermentation to stabilize methane production at a high OLR and to shorten the startup period by accelerating microbial growth (Sasaki et al. 2010b, 2011a). In addition, a BER containing CFT (BER + CFT) was exploited to have characteristics of a packed-bed and bioelectrochemical systems (Sasaki et al. 2011b). By introducing the electrochemical system to the methanogenic reactor, the BER + CFT (250-mL working volume) attained stable methane production at an OLR of 27.8 g-dichromate chemical oxygen demand (CODcr) · L^−1^ · day^−1^, using a substrate that mainly contained an artificial garbage slurry (AGS). In addition, effect of electrochemical regulation in BER + CFT was clarified by comparing BER + CFT with non-BER + CFT (without electrochemical regulation) (Sasaki et al. 2011b).

A scaled-up BER + CFT (2.4-L working volume) was operated at laboratory scale using a cylindrical type of reactor to apply BER + CFT to the usual methane fermentation system because the cylindrical type of reactor is utilized in anaerobic digestion (Angenent et al. 2002; Tatara et al. 2008). The cylindrical type of BER + CFT has a specific configuration in which the working chamber surrounds the counter chamber, and those 2 chambers are separated by a proton exchange membrane. Sewage sludge was applied to a cylindrical type of BER + CFT in our previous research and a scaled-up BER + CFT also showed the effect of electrochemical regulation (Sasaki et al. 2013). However, given that the concentration of organic matter in the sewage sludge was relatively low, high OLR operation was difficult. A garbage slurry including high percentage of solid fraction has not yet been applied to the cylindrical type of BER + CFT.

In the present study, the aim was to operate at high OLR conditions and investigate the reactor performance by using AGS (10% dog food slurry) as a model of municipal solid waste and microbial community in the cylindrical type of BER + CFT. High OLR operation is important to demonstrate the potential for this technology in the efficient treatment of garbage slurry with a high percentage of solid content. Microbial communities on the working electrode and in the suspended fraction were quantitatively and qualitatively analyzed by 16S rRNA gene-based techniques such as real-time PCR assay and terminal restriction fragment length polymorphism (T-RFLP) analysis.

## Materials and methods

### Feed material

The AGS was prepared from 100 g of a commercial dog food (VitaOne, Nihon Pet Food, Tokyo, Japan) suspended in 1.0 L of sterilized water. The physicochemical characteristics of the AGS were as follows: total CODcr, 134.1 g-CODcr · L^−1^; CODcr of supernatant, 8.4 g-CODcr · L^−1^; suspended solid (SS), 54.0 g · L^−1^; and volatile suspended solid (VSS), 45.5 g · L^−1^.

### BER + CFT

**Figure 1:**
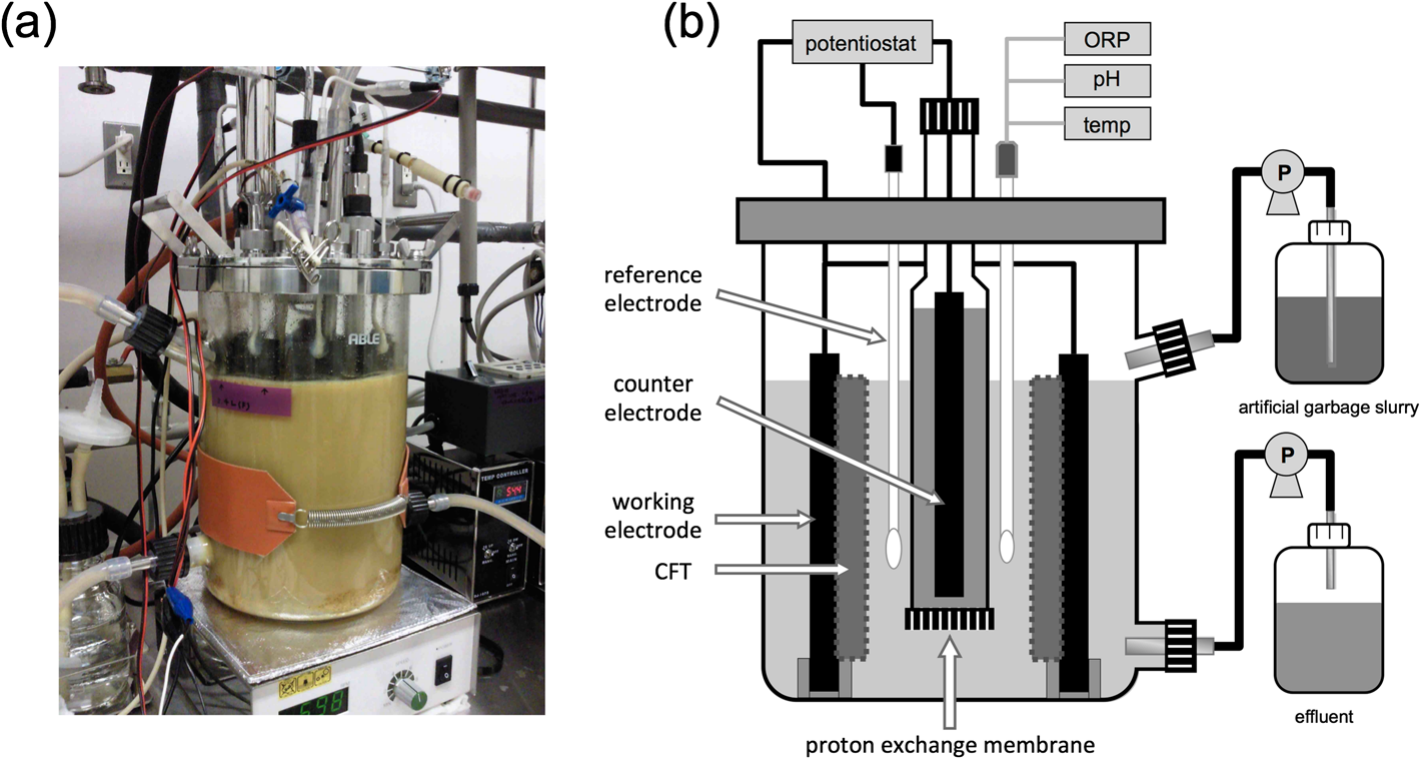
**Photograph of the BER + CFT (a) and schematic diagrams of the BER + CFT (b).** ORP: oxidation-reduction potential.

The BER + CFT was constructed from a glass vessel (4.0-L capacity) with several sampling ports and flow-line nozzles (Figure 1). The BER + CFT was separated into 2 chambers by a proton exchange membrane (Nafion N117, Dupont Co., Delaware, USA). CFT (150 × 164 × 3 mm) was adhered to the inside of a carbon plate (150 × 35 × 6 mm), and 8 carbon plates holding CFT were used to equip a cathodic working chamber (2.4-L working volume) as the cathodic working electrode and supporting material. To control the potential on the cathodic working electrode, an Ag/AgCl reference electrode was inserted into the working chamber. The anodic counter chamber filled with 100-mM NaCl (180 mL) was installed in the center of a reactor, and a columnar carbon bar (height = 150 mm; radius = 4 mm) was used as an anodic counter electrode.

Cathodic and anodic electrodes, and the reference electrode placed in the cathodic working chamber were connected to a potentiostat (PS-08, Tohogiken, Japan). Potential on the cathodic working electrode was regulated at −0.8 V (vs. Ag/AgCl) because we previously reported that stable methane production and decomposition of garbage slurry at a high OLR were observed in a small-scale thermophilic bioelectrochemical methanogenic reactor (250-mL working volume) regulated at −0.8 V (Sasaki et al. 2010b). All the voltages reported in this paper are with respect to the Ag/AgCl reference electrode (+201 mV vs. standard hydrogen electrode). The oxidation-reduction potential was monitored, and pH and temperature were controlled by discrete control units (DJ-1023, DJ-10083, and DJ-1073; ABLE, Tokyo, Japan).

### Operation of the BER + CFT

Seed sludge was collected from a packed-bed thermophilic methanogenic digester degrading AGS, operated at an HRT of 20 days, in which stable gas production was observed. The seed sludge (2.4 L) was put into the cathodic working chamber that was then sealed with a top plate, electrodes, sensors, and stainless pipes, and the fermentation broth was thoroughly mixed using a magnetic stirrer. The initial anaerobic condition was established by replacing the gas phase with nitrogen gas. The temperature of the culture was maintained at 55°C. The operation of the cathodic working chamber was conducted as follows: the predetermined volume of fermentation broth was discharged, and the same amount of fresh AGS was added by using a timer and peristaltic pumps (RP2000, EYELA, Tokyo, Japan). The pH was adjusted to approximately 7.2, with 1.0-N NaOH, throughout the experiment.

**Figure 2:**
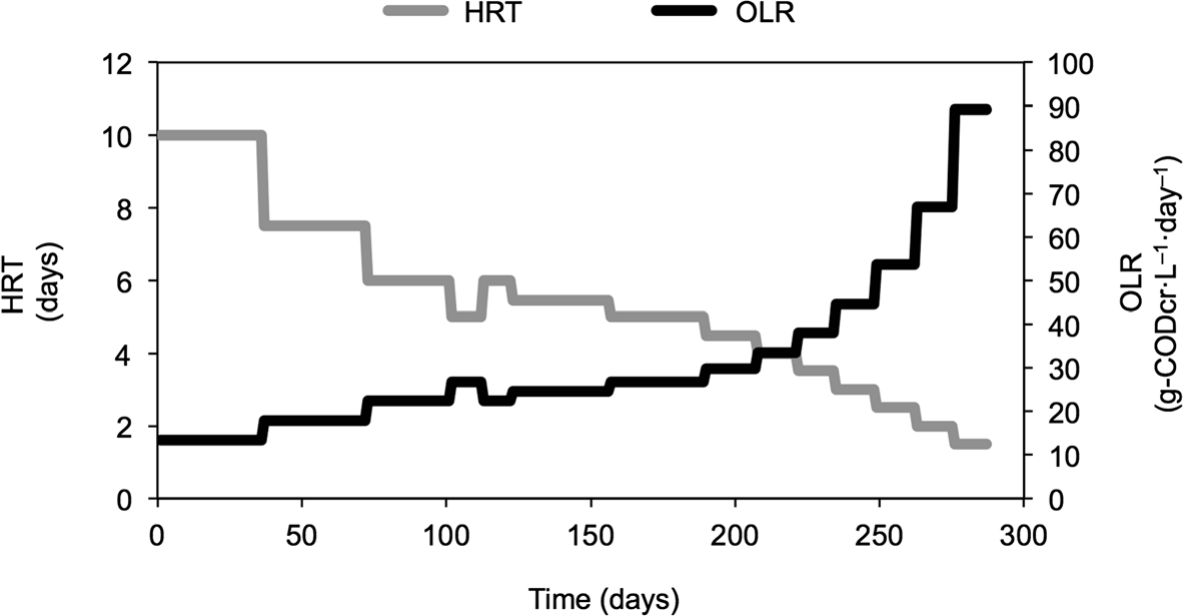
**Time courses of HRT (gray line) and OLR (black line) in working chambers of the cathodic BER + CFT.** The operation was carried out 285 days from the OLR (HRT) of 13.4 (10 days) to 89.3 g-CODcr · L^−1^ · day^−1^ (1.5 days).

The time course for the changes in the HRT and OLR in the cathodic working chamber are summarized in Figure 2. The OLR was increased in a stepwise manner by decreasing the HRT after operation during 3 or more times of HRT. The suspended fractions in the reactors were collected for analysis as the end point for each HRT. In addition, the biomass retained by the working electrode was sampled at day 275 (HRT of 2.0 days). To collect retained biomass, the sample was vigorously vortexed in phosphate-buffered saline, after which any remaining biomass was scraped off.

### Analyses of reactor performance

The gas production rate was measured periodically by water displacement with a graduated cylinder. The CH_4_, CO_2_, and H_2_ contents of the produced gas were measured with a gas chromatograph equipped with a thermal conductivity detector (GC390B, GL Sciences, Tokyo, Japan) and a stainless steel column packed with active carbon (30/60 mesh; GL Sciences). The suspended fractions in the reactors were collected for analyses of physicochemical parameters at approximately 3-day intervals. The CODcr was determined by using the dichromate method with a COD analyzer (DR/800, Hach, Loveland, CO, USA). To determine the total SS, 2–10 mL of the suspended fraction was passed through the membrane (glass fiber membrane: 0.45 μm, 47 mm; Toyo Roshi Kaisha, Ltd., Tokyo, Japan), after which the membrane was weighed after drying at 105°C for 120 min. VFAs (formate, acetate, propionate, and butyrate) were measured using high-pressure liquid chromatography equipped with an organic acid analysis system (TSK-GEL OApak-A and OApak-P, Tosoh, Tokyo, Japan).

### DNA extraction and purification

Biomasses in the suspended fraction and retained on the working electrode of the reactors were centrifuged at 5,000 × *g*, and the resultant pellets were suspended in Tris-EDTA buffer (100-mM Tris–HCl and 40-mM EDTA, pH 8.0). DNA was extracted from the pellets using repeated bead beating in the presence of sodium dodecyl sulfate and phenol-chloroform-isoamyl alcohol (25:24:1), after which the DNA was purified using a QIAamp DNA Micro Kit (Qiagen, Tokyo, Japan), as described previously (Sasaki et al. 2009).

### Quantitative PCR analysis

Real-time PCR was performed using a LightCycler 1.5 (Roche Diagnostics, Tokyo, Japan) and LightCycler TaqMan Master (Roche Diagnostics), as previously described (Sasaki et al. 2010a). Primer sets of S-P-MArch-0348-S-a-17 and S-D-Arch-0786-A-a-20 were used with the double-dye probe March-0515 for measuring the copy number of 16S rRNA from methanogenic archaea (Sawayama et al. 2004). The primer set Uni340F and Uni806R were used with the double-dye probe Uni516F for prokaryotes (Takai and Horikoshi 2000).

### T-RFLP analysis

PCR amplification was performed using AmpliTaqGold (Applied Biosystems, Tokyo, Japan). The primer sets used were Ba27f (*Escherichia coli* positions 8–27) and Ba907r (*E. coli* positions 907–926) for the domain *Bacteria* (Lueders and Friedrich 2002), or Ar109f (*E. coli* positions 109–125) and Ar912rt (*E. coli* positions 912–934) for the domain *Archaea* (Lueder et al. 2004). T-RFLP analysis was conducted as previously described (Sasaki et al. 2010a).

## Results

### Reactor performance in BER + CFT

**Figure 3:**
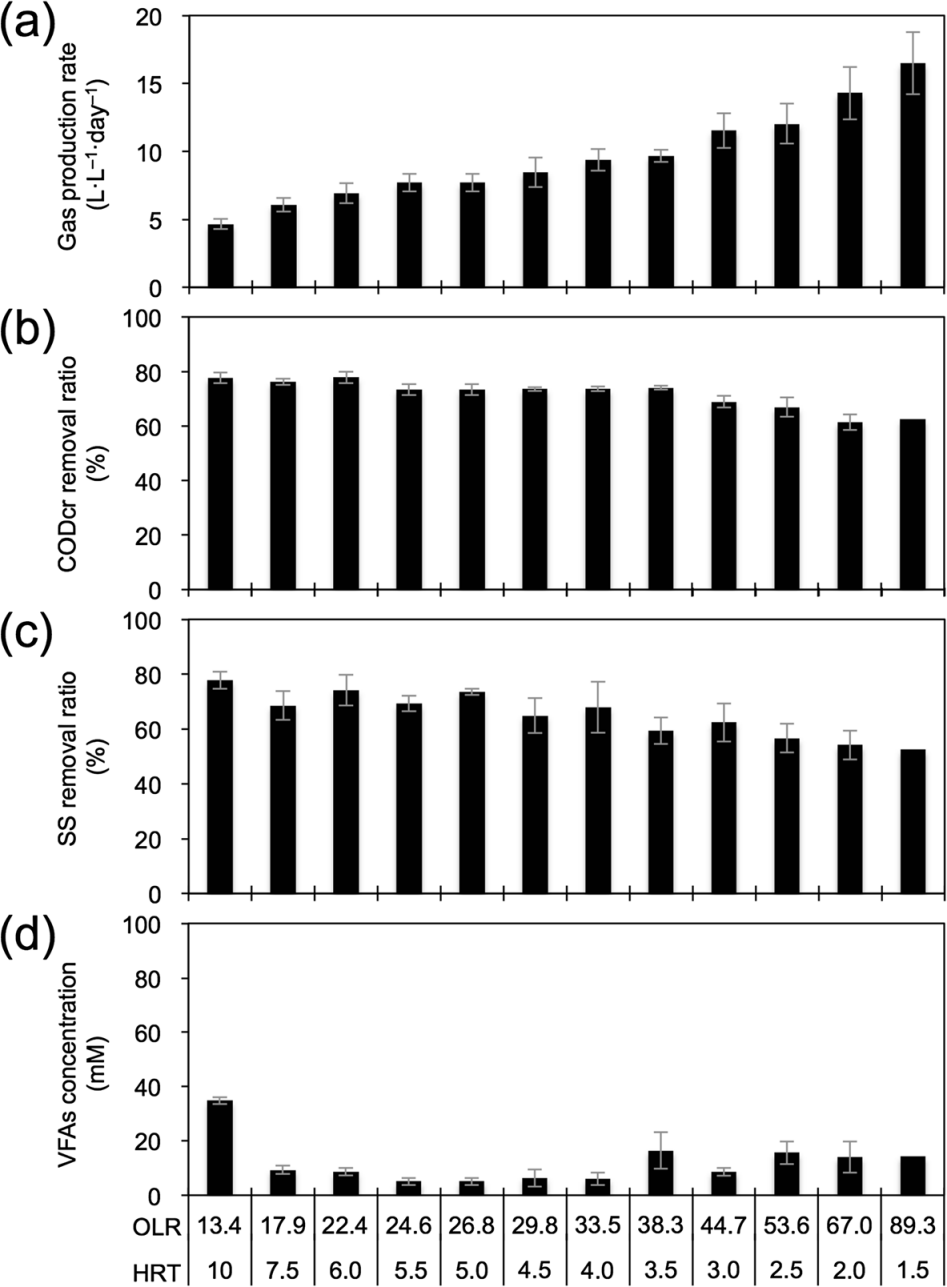
**Gas production rate (a), CODcr removal ratio (b), SS removal ratio (c), and VFAs concentration (d) in the cathodic working chambers in the BER + CFT.** Error bars show standard deviations for the mean of several days’ performance in the same OLR operation. The performance was calculated from the OLR (HRT) of 13.4 (10 days) to 89.3 g-CODcr · L^−1^ · day^−1^ (1.5 days).

The mean values of reactor performance at OLRs from 13.4 (HRT of 10 days) to 89.3 g-CODcr · L^−1^ · day^−1^ (HRT of 1.5 days) are summarized in Figure 3. The gas production rate in the BER + CFT increased as the OLR increased (Figure 3a). The CH_4_ contents in the produced gas were constant throughout the operation, and the contents of CH_4_ and CO_2_ were approximately 60% and 40%, respectively (data not shown). H_2_ gas was not detected in the produced gas. Finally, the mean values of the gas production rate (volume of CH_4_ gas) and CH_4_ content at 89.3 g-CODcr · L^−1^ · day^−1^ (HRT of 1.5 days) were 16.5 L · L^−1^ · day^−1^ (9.37 L-CH_4_ · L^−1^ · day^−1^) and 56.8%, respectively, under standard conditions (Table 1). The CODcr and SS removal efficiencies were higher than 60% (Figure 3b) and 50%, respectively (Figure 3c), until the end point in the operation. Acetate, propionate, and butyrate were detected as VFAs throughout the operation periods (8.3, 5.0, and 0.9 mM at an OLR of 89.3 g-CODcr · L^−1^ · day^−1^, respectively). The VFA concentrations were less than 20 mM at all OLRs, except 13.4 g-CODcr · L^−1^ · day^−1^ (Figure 3d).

**Table 1:** Reactor performances in thermophilic BER + CFT and previous reactors treating garbage waste under the maximum OLR conditions

	**Treating waste**	**OLR (g-CODcr · L**^**−1**^ **· day**^**−1**^**)**	**HRT (days)**	**CODcr removal ratio (%)**	**VTS removal ratio (%)**	**SS removal ratio (%)**	**VSS removal ratio (%)**	**Gas production rate**^**a **^**(L · L**^**−1**^ **· day**^**−1**^**)**	**Methane content (%)**	**CODcr recovery**^**b **^**(%)**	**References**
Cylindrical BER + CFT	10% AGS	89.3	1.5	62.5	-	52.5	50.9	16.5 ± 3.4	56.8	83.9	In this study
		53.6	2.5	67.0 ± 3.5	-	56.6 ± 5.3	51.7 ± 4.9	12.1 ± 1.5	58.2 ± 1.7	117.1 ± 13.9	
Packed-bed reactor	1.5% AGS + 1.0% milled paper	53.6	0.9	67.0	-	-	67.0	11.4	60.0	56.2	Ueno et al. (2007)
Small-scale BER + CFT (−0.8 V)	10% AGS + 0.08% ground rice straw	27.8	4.4	58.0 ± 4.9	-	38.9 ± 1.9	-	7.9 ± 0.7	55.0 ± 3.2	86.5 ± 2.6	Sasaki et al. (2011b)
CFSTR	10% artificial kitchen waste	20.4	11.0	89.0		-	-	-	-	82.0	Park et al. (2008)
UAF reactor	Synthetic garbage + swine manure	17.6	9.2	-	78.0	-	-	8.7	50.0	-	Liu et al. (2009)
CFSTR	2% AGS	6.25	4.0	65.8 ± 2.8	-	61.8 ± 5.6	-	1.8 ± 0.1	80.0	-	Sasaki et al. (2011c)

^a^Mean values under standard temperature and pressure at these OLRs are shown.

^b^CODcr recovery = [(CODcr of the fermentation broth) + (CODcr of CH_4_)] / Influent CODcr.

VTS: volatile total solid, SS: suspended solid, VSS: volatile suspended solid, UAF reactor: upflow anaerobic filter reactor, CFSTR: continuously flow-stirred tank reactor, AGS: artificial garbage slurry (dog food slurry).

### Diversity of microbial community

**Figure 4:**
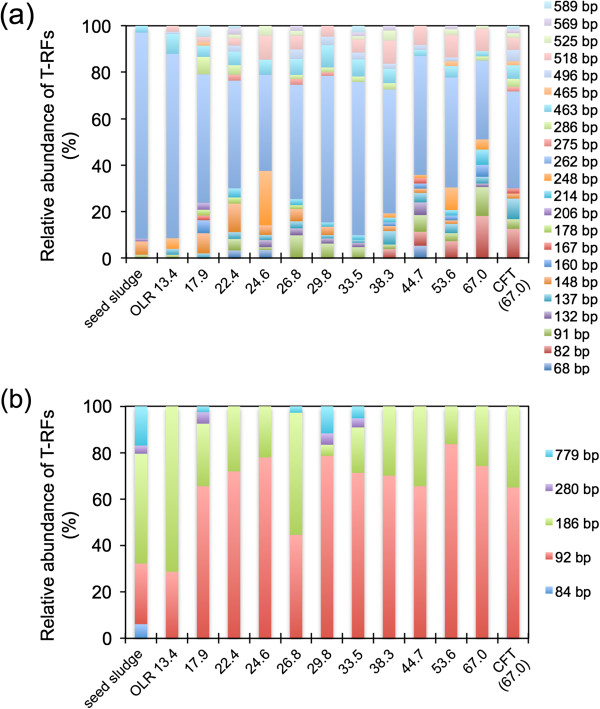
**Community dynamics of bacteria (a) and archaea (b), determined by T-RFLP analysis in the BER + CFT.** The relative abundances of T-RF generated from DNA samples in the seed sludge, in the suspended fraction at the OLR of 13.4, 17.9, 22.4, 26.8, 29.8, 33.5, 38.3, 44.7, 53.6, and 67.0 g-CODcr · L^−1^ · day^−1^ and in the retained fraction at the OLR of 67.0 g-CODcr · L^−1^ · day^−1^.

To investigate the bacterial and archaeal community structure, T-RFLP analyses were performed for the suspended fraction (fermentation broth) at several points and for the retained fraction in the CFT at an OLR of 67.0 g-CODcr · L^−1^ · day^−1^ (Figure 4). From the analysis of bacterial T-RFLP in the suspended fraction, 22 terminal restriction fragments (T-RFs) were detected. A T-RF of 262 bp was dominant throughout the operation period (34.0–79.4%). In comparison with the bacterial community in the retained fraction with the CFT, no remarkable differences were observed in the suspended fraction and a T-RF of 262 bp was also dominant (41.7%) in the retained fraction.

In the analysis of archaeal T-RFLP, only 5 of the T-RFs (84, 92, 186, 280, and 779 bp) were detected. T-RFs of 92 and 186 bp were predominant. At an OLR of 67.0 g-CODcr · L^−1^ · day^−1^, the archaeal T-RFLP profiles were similar between the suspended and retained fractions.

### Quantitative analysis of prokaryotes and methanogenic archaea

Real-time quantitative PCR analysis of the 16S rRNA gene for prokaryotes and methanogenic archaea was conducted in the suspended fraction at several time points of the OLR conditions and the retained fraction to CFT at an OLR of 67.0 g-CODcr · L^−1^ · day^−1^ (Table 2). In the suspended fractions, prokaryotic and methanogen copy numbers were 8.7 × 10^12^–1.5 × 10^13^ copies · reactor^−1^ and 2.3 × 10^12^–4.7 × 10^12^ copies · reactor^−1^, respectively. Ratios of methanogens to prokaryotes in the suspended fraction were 24.2–31.2% at OLRs from 13.4 to 67.0 g-CODcr · L^−1^ · day^−1^. However, the ratio of methanogens in the retained fraction to CFT was higher, that is, 52.3% at an OLR of 67.0 g-CODcr · L^−1^ · day^−1^. The ratio of methanogens in the retained fraction to those in the total fractions (suspended fraction plus retained fraction) was 27.7% at an OLR of 67.0 g-CODcr · L^−1^ · day^−1^.

**Table 2:** **16S rRNA gene copy numbers of prokaryotes and methanogens in the suspended fraction (OLRs of 13.4, 33.5, 38.0, 53.6, and 67.0 g-CODcr · L**^**−1**^ **· day**^**−1**^**) and retained fraction on the CFT (OLR of 67.0 g-CODcr · L**^**−1**^ **· day**^**−1**^**) of the BER + CFT**

**OLR (g-CODcr · L**^**−1**^ **· day**^**−1**^**)**	**Prokaryotes (copies · reactor**^**−1**^**)**	**Methanogens (copies · reactor**^**−1**^**)**	**Ratio of methanogens**^**a **^**(%)**
**Suspended fraction**			
13.4	(9.6 ± 0.4) × 10^12^	(2.3 ± 0.3) × 10^12^	24.2 ± 4.0
33.5	(8.7 ± 0.8) × 10^12^	(2.6 ± 0.1) × 10^12^	29.6 ± 3.7
38.0	(1.3 ± 0.1) × 10^13^	(4.0 ± 0.5) × 10^12^	31.2 ± 6.7
53.6	(1.2 ± 0.2) × 10^13^	(3.2 ± 0.2) × 10^12^	27.1 ± 6.7
67.0	(1.5 ± 0.1) × 10^13^	(4.7 ± 0.1) × 10^12^	31.2 ± 2.1
**Retained fraction**			
67.0	(3.4 ± 0.5) × 10^12^	(1.8 ± 0.1) × 10^12^	52.3 ± 9.6

^a^Proportion of 16S rRNA gene copy number of methanogens to that of prokaryotes.

## Discussion

### Utility of the cylindrical BER + CFT

Various methane fermentation systems have so far been examined in the processing of organic garbage wastes (Table 1). Ueno et al. (2007) operated at quite high OLR, in a thermophilic packed-bed system that packed CFT, and used AGS plus milled paper as substrate, attaining 11.4 L · L^−1^ · day^−1^ at an OLR of 53.6 g-CODcr · L^−1^ · day^−1^. By using an OLR of 53.6 g-CODcr · L^−1^ · day^−1^ in this BER + CFT, the reactor in this study and the packed-bed reactor of Ueno et al. (2007) showed nearly the same values (Table 1). However, the cylindrical BER + CFT in this study could attain 16.5 L · L^−1^ · day^−1^ at an OLR of 89.3 g-CODcr · L^−1^ · day^−1^, which is higher than about 12.5 L · L^−1^ · day^−1^ at an OLR of about 85.0 g-CODcr · L^−1^ · day^−1^, attained by the packed-bed system of Ueno et al. (2007). Stable reactor performance was observed from 53.6 to 89.3 g-CODcr · L^−1^ · day^−1^ in the cylindrical BER + CFT, although the reported packed-bed reactor deteriorated in these OLR conditions. Therefore, the higher OLR operation was achieved using AGS including high percentage of solid fraction in the BER + CFT by introducing a bioelectrochemical system.

### Microbial roles in the cylindrical BER + CFT

From the previous results of T-RFLP and clone analysis (Sasaki et al. 2010b, dominant T-RF of 262 bp had 99% sequence similarity with the 16S rRNA gene of *Defluviitoga tunisiensis (FR850164; Ben Hania et al. *2012). *D. tunisiensis* belongs to the thermophilic phylum Thermotoga and utilizes many kinds of substrates (e.g., saccharides and cellulose). In the previous results, this T-RF was also dominant in the continuously flow-stirred tank reactor (CFSTR) (Sasaki et al. 2011c) or thermophilic BER degrading AGS (Sasaki et al. 2010b). It has been suggested that the microorganism related to this T-RF is playing an important role in degrading organic materials in garbage wastes (Ben Hania et al. 2012; Sasaki et al. 2011c).

From the previous results of T-RFLP and clone analysis (Sasaki et al. 2010b, 2011b, predominant archaeal T-RFs of 92 and 186 bp were related to the hydrogenotrophic methanogen, *Methanothermobacter thermautotrophicus* (AY196660; Wasserfallen et al. 2000), and the aceticlastic methanogen, *Methanosarcina thermophila* (M59140; Rouvière et al. 1992). The remaining T-RFs of 84, 280, and 779 bp were related to the hydrogenotrophic methanogens *Methanoculleus thermophilus* (EF118904; Spring et al. 2005), *Methanothermobacter thermautotrophicus* (Wasserfallen et al. 2000), and *Methanobacterium formicicum* (HQ591420; Sousa et al. 2007), respectively. It has been shown that cathodic reaction by BER increased the growth and methanogenesis of hydrogenotrophic methanogen (Sasaki et al. 2012a; Hirano et al. 2013). Hydrogenotrophic methanogen retained in electron-conductive CFT would be affected by electrochemical regulation. In addition, *M. thermophila*-related microorganisms could become dominant in CFT due to their aggregating nature (Sasaki et al. 2007). From the T-RFLP results, the archaeal (methanogenic) community structure did not show an appreciable succession by changing the OLR conditions through the operating period. Hori et al. (2006) reported that the instability of reactor performance affected the structure of the methanogenic microbial community in the CFSTR, degrading a synthetic substrate after the reactor deteriorated. Therefore, a stable methanogenic community structure would be important for the stable operation of cylindrical BER + CFT under high OLR conditions.

From the results of quantitative analysis of methanogenic archaea, methanogens were highly retained to CFT by attaching directly onto the cathodic electrode, in agreement with previous results (Sasaki et al. 2011b). The ratios of the methanogens in the suspended and retained fractions were comparatively higher than those in the small-scale (250-mL working volume) BER + CFT (ratio of methanogens to prokaryotes in suspended and retained fractions: 15.7–21.3% and 34.3–36.9%, respectively; Sasaki et al. 2011b). Maintaining methanogens within the reactor would be favorable for operating at high OLR. Also taking into consideration the T-RFLP analysis results, hydrogenotrophic and aceticlastic methanogens could stably grow in CFT. Stable retention of an aceticlastic methanogen would contribute to efficient removal of acetate, thereby preventing acetate accumulation (Sasaki et al. 2007). Hydrogenotrophic methanogens retained in the electron-conductive CFT would work well for hydrogen removal to increase degradation activity of hydrolytic bacteria because syntrophic degradation accelerated the removal of protein or cellulose (Tang et al. 2005; Sasaki et al. 2012a, 2012b).

### Current in the cylindrical BER + CFT

In BER + CFT, the oxidation-reduction potential of the fermentation broth was approximately −0.45 V (data not shown). The settled potential (−0.8 V) was lower than that in the fermentation broth. Therefore, negative current densities were observed in BER + CFT throughout the operation. These results show that the cathodic reaction occurred on the working electrode in the BER + CFT regulated at −0.8 V. The electric current values were nearly similar through the operating period. Here, we discuss the possibility of direct methane production from current reported according to the following reaction (Cheng et al. 2009):

$${\mathrm{CO}}_{2}+{8H}^{+}+{8e}^{-}\to {\mathrm{CH}}_{4}+{2H}_{2}O$$

Eight electrons (771,882 C, 8 times the Faraday constant) are required to produce 1 mol of CH_4_. In the BER + CFT at −0.8 V, the mean current density was −0.21 A · m^−2^ at an OLR of 89.3 g-CODcr · L^−1^ · day^−1^. Therefore, 763.8 C (8.84 × 10^−3^ A × 86,400 s) of the mean charges is used in 1 day per the total working electrode (4.2 × 10^−2^ m^2^). If all of the current is used to produce CH_4_, then the current corresponds to only 9.24 × 10^−3^ L-CH_4_ · L^−1^ · day^−1^. However, in the BER + CFT, an additional 9.36 L-CH_4_ · L^−1^ · day^−1^ was generated [(9.37 − 9.24 × 10^−3^) L-CH_4_ · L^−1^ · day^−1^]. Therefore, almost all of the CH_4_ would come from the substrate, suggesting that effects other than direct methane production from the current work for efficient operation of the BER + CFT.

### Effectiveness of electric energy

The input electric energy was compared with the output of the evolved CH_4_ in the stable operation of the BER + CFT regulated at −0.8 V at an OLR of 89.3 g-CODcr · L^−1^ · day^−1^. At this OLR (HRT of 1.5 days), absolute values of the mean current densities and voltages between the working electrode and counter electrode were 0.21 A · m^−2^ and 1.88 V. As a result, 16.6 mW · reactor^−1^ · day^−1^ (0.6 kJ · L^−1^ · day^−1^) of the input energies were consumed per total CFT (4.2 × 10^−2^ m^2^). The mean value of CH_4_ gas was 9.37 L-CH_4_ · L^−1^ · day^−1^, and from the reaction enthalpy (Δr*Hº*) for the combustion of CH_4_ gas, which is −890.71 kJ · mol^−1^ under standard conditions (Dean 1999), CH_4_ production corresponded to 372.6 kJ · L^−1^ · day^−1^ [(9.37 L-CH_4_ · L^−1^ · day^−1^ / 22.4 L) × 890.71 kJ · mol^−1^]. It was revealed that much higher output energy as CH_4_ was produced than input energies.

It has been predicted that electric input energy is used to control the redox potential in the fermentation broth, which is optimal for a complex microbial community that includes bacteria and/or methanogens. A syntrophic relationship between bacteria and methanogens was often reported in methanogenic microbial communities (Stams and Plugge 2009; Sasaki et al. 2012b). To understand the effect of the electrochemical reaction on the microbial relationship, information on the isolated microorganisms would be required from now on. We succeeded in stably operating the scaled-up cylindrical BER + CFT to treat the model garbage waste at high OLR. The scaled-up cylindrical BER + CFT can be utilized for construction of sustainable society recycling of municipal solid wastes.

## Competing interests

The authors declare that they have no competing interests.
